# Supplementary material for: Parental Willingness and Associated Factors of Pediatric Vaccination in the Era of COVID-19 Pandemic: A Systematic Review and Meta-Analysis
Source: Vaccines (Basel). 2022 Sep 2;10(9):1453. doi: 10.3390/vaccines10091453 (PMC9506252; doi:10.3390/vaccines10091453)
Supplement: Supplementary file 1 [file vaccines-10-01453-s001.zip › vaccines-1865152-supplementary.pdf]

Supplementary Material

Table S1. Quality assessment of the included studies.

No	Included paper	Methodological items for rating (1–4)*									
		Abstract & title	Introduction and aims	Method and data	Sampling	Data analysis	Ethics and bias	Results	Transferability (generalizable)	Implications and usefulness	Total score
1	Wang et al., 2022	4	4	3	4	3	4	3	3	3	31
2	Sinuraya et al., 2022	3	3	3	2	3	2	3	2	3	24
3	Powelson et al., 2022	4	4	2	2	3	3	3	2	3	26
4	Özer et al., 2022	4	4	3	3	3	3	3	3	3	29
5	Olagoke et al., 2022	3	3	3	2	3	2	3	2	3	24
6	Hill et al., 2022	3	2	3	2	3	2	3	2	3	23
7	He et al., 2022	4	4	3	2	3	2	3	2	3	26
8	Gjini et al., 2022	3	3	3	3	3	3	3	3	3	27
9	Du et al., 2022	4	4	3	4	3	4	3	3	3	31
10	Çağ et al., 2022	3	3	3	4	2	3	2	3	2	25
11	Zhou et al., 2021	3	3	3	3	3	3	3	3	3	27
12	Seiler et al., 2021	3	3	3	3	3	3	3	3	2	26
13	Salawati et al., 2021	4	4	3	4	3	4	3	3	3	31
14	Hou et al., 2021	4	4	3	4	3	4	3	3	3	31
15	Goldman et al., 2021	3	3	3	4	3	3	3	3	3	28
16	Beatty & Villwock, 2021	3	2	3	2	2	2	2	2	2	20
17	Baghdadi et al., 2021	3	3	3	3	2	3	2	3	2	24
18	Al-Nafeesah et al., 2021	3	2	3	4	2	2	2	3	2	23
19	Aldakhil et al., 2021	3	3	3	2	3	2	3	2	3	24
20	Sokol & Grummon, 2020	3	3	3	4	3	4	3	3	2	28

* We used the tool developed by Hawker et al to assess quality of the studies. The tool covers nine domains: 1) abstract and title (did they provide a clear description of the study), 2) introduction and aims (was there a good background and clear statement of the aims of the research), 3) method and data (is the method appropriate and clearly explained), 4) sampling (was the sampling strategy appropriate to address the aims), 5) data analysis (was the description of the data analysis sufficiently rigorous), 6) ethics and bias (how ethical issues been addressed, and what has necessary ethical approval gained? Has the relationship between researchers and participants been adequately considered), 7) results (is these a clear statement of the findings), 8) transferability and generalizability (are the findings of this study transferable/generalizable to a wider population), and 9) implications and usefulness (how important are these findings to policy and practice). These domains are rated from 1=very poor, 2=poor, 3=fair, to 4=good. Criteria of ratings followed the statements listed in Appendix D (p. 1296-1297) of Hawker, S., Payne, S., Kerr, C., Hardey, M., & Powell, J. (2002). Appraising the evidence: Reviewing disparate data systematically. Qualitative Health Research, 12(9), 1284-1299. doi:10.1177/1049732302238251.

Table S2. Categories and associated factors of parental willingness to vaccinate their children.

No	Ref	Associated Factors of Parental Willingness to vaccinate their children														
		**Older children**	**Higher education level**	**Health care workers**	**Regional differences**	**Confidence in vaccines**	**Concerns about side effects**	**Increased number of Children**	**Higher income**	**Influenza history of Children**	**Influenza history of parents**	**Children with chronic and or other diseases**	**Trust in health care information Source**	**History of COVID-19 infection in family**	**COVID-19 vaccine uptake/intention among parents**	**Level of analysis**
**Childhood/routine vaccination**																
1	Sinuraya et al., 2022	-	↑	↑	-	↑	-	-	-	-	-	-	-	-	-	**Multivariable**
2	Baghdadi et al., 2021	-	-	-	-	-	-	-	-	-	-	-	↑	-	-	**Multivariable**
3	Al-Nafeesah et al., 2021	-	-	-	**living in Riyadh or the Western regions↓**	↑	-	↓	-	-	-	-	-	-	-	**Multivariable**
4	Aldakhil et al., 2021	-	↑	-	-	↑	↓	-	-	-	-	-	↑	-	-	**Multivariable**
5.	Wang et al., 2022								*NA*							
6.	He et al., 2022								*NA*							
7.	Gjini et al., 2022								*NA*							
8.	Çağ et al., 2022								*NA*							
**Influenza vaccination**																
9.	Özer et al., 2022	-	-	-	-	-	-	-	-	-		↑	-	↑	-	**Multivariable**
10.	Hill et al., 2022	-	-	-	-	-	-	-	-	↑	-	-	-	-	↑	**Multivariable**
11.	Zhou et al., 2021								*NA*							
12.	Seiler et al., 2021	-	-		**Latin Switzerland↑German Switzerland ↓**	-	-	-	-	-	-	↑	-	-	-	**Multivariable**
13.	Salawati et al., 2021	-	↑	↑	-	-	-	-	↑	↑	-	↑	↑	-	↑	**Multivariable**
14.	Hou et al., 2021	↓	↑	-	-	-	-	-	-	-	-	-	-	-	-	**Multivariable**
15.	Goldman et al., 2021	-	↑	-	-	-	-	-	-	↑	↑	↑	-	-	-	**Multivariable**

No	Ref	Associated Factors of Parental Willingness to vaccinate their children														
		**Older children**	**Higher education level**	**Health care workers**	**Regional differences**	**Confidence in vaccines**	**Concerns about side effects**	**Increased number of Children**	**Higher income**	**Influenza history of Children**	**Influenza history of parents**	**Children with chronic and or other diseases**	**Trust in health care information Source**	**History of COVID-19 infection in family**	**COVID-19 vaccine uptake/intention among parents**	**Level of analysis**
16.	Du et al., 2022								*NA*							
17	Beatty & Villwock, 2021								*NA*							
18	Sokol & Grummon, 2020								*NA*							
**Human papillomaviruses (HPV) vaccination**																
19	Olagoke et al., 2022								*NA*							
**Pneumococcal conjugate vaccination (PCV)**																
20	Powelson et al., 2022								*NA*							
Positive association[a]		**0/1**	**5/5**	**2/2**	**1/3**	**3/3**	**0/1**	**0/1**	**1/1**	**3/3**	**1/1**	**4/4**	**3/3**	**1/1**	**2/2**	
Negative association[b]		**1/1**	**0/5**	**0/2**	**2/3**	**0/3**	**1/1**	**1/1**	**0/1**	**0/3**	**0/1**	**0/4**	**0/3**	**0/1**	**0/2**	

↑ more likely to vaccinate. ↓ less likely to vaccinate. - not investigated. NA not applicable. [a] Number of studies with a positive significant association (p-value < 0.05) between the predictor and parents' willingness to vaccinate their children/total number of studies examined the predictor. [b] Number of studies with a negative significant association (p-value < 0.05) between the predictor and parents' willingness to vaccinate their children/total number of studies examined the predictor.

Table S3. Categories and associated factors of parental hesitancy to vaccinate their children.

No	Ref	**Associated Factors of Parental hesitancy to vaccinate their children**													
		**Older parents**	**Higher education level**	**Fathers**	**Ethnicity**	**Low Confidence in vaccines**	**Concerns about side effects**	**Lower health status of parents**	**Higher income**	**Health care workers**	**Influenza history of parents**	**Children with chronic and or other diseases**	**Low trust in health care information Source**	**COVID-19 vaccine uptake/intention among parents**	**Level of analysis**
**Childhood/routine vaccination**															
1	Wang et al., 2022	-	-	↑	-	-	-	↓	-	-	-	-	-	-	**Multivariable**
2	He et al., 2022	↓	↓	-	**Hispanic ethnicity and other multiple race↑**	-	-	-	↓	-	-	-	-	-	**Multivariable**
3	Gjini et al., 2022	-	-	-	-	↑	-	-	-	-	-	-	↑	-	**Univariable**
4	Aldakhil et al., 2021	-	↑	-	-	↑	↑	-	-	-	-	-	↑	-	**Multivariable**
5.	Çağ et al., 2022	-	↑	-	-	-	-	-	-	↓	↓	-	↑	↓	**Multivariable**
6.	Sinuraya et al., 2022								*NA*						
7.	Baghdadi et al., 2021								*NA*						
8.	Al-Nafeesah et al., 2021								*NA*						
**Influenza vaccination**															
9.	Du et al., 2022	-	-	-	-	↑	-	-	-	-		↓	-	-	**Multivariable**
10.	Zhou et al., 2021								*NA*						
11.	Özer et al., 2022								*NA*						
12.	Hill et al., 2022								*NA*						
13.	Seiler et al., 2021								*NA*						
14.	Salawati et al., 2021								*NA*						

No	Ref	Associated Factors of Parental hesitancy to vaccinate their children													
		Older parents	Higher education level	Fathers	Ethnicity	Low Confidence in vaccines	Concerns about side effects	Lower health status of parents	Higher income	Health care workers	Influenza history of parents	Children with chronic and or other diseases	Low trust in health care information Source	COVID-19 vaccine uptake/intention among parents	Level of analysis
15.	Hou et al., 2021								*NA*						
16.	Goldman et al., 2021								*NA*						
17.	Beatty & Villwock, 2021								*NA*						
18.	Sokol & Grummon, 2020								*NA*						
**Human papillomaviruses (HPV) vaccination**															
19.	Olagoke et al., 2022								*NA*						
**Pneumococcal conjugate vaccination (PCV)**															
20.	Powelson et al., 2022								*NA*						
Positive association[a]		**0/1**	**2/3**	**1/1**	**1/1**	**3/3**	**1/1**	**0/1**	**0/1**	**0/1**	**0/1**	**0/1**	**3/3**	**0/1**	
Negative association[b]		**1/1**	**1/3**	**0/1**	**0/1**	**0/3**	**0/1**	**1/1**	**1/1**	**1/1**	**1/1**	**1/1**	**0/3**	**1/1**	

↑ more likely to vaccinate. ↓ less likely to vaccinate. - not investigated. NA not applicable. [a] Number of studies with a positive significant association (p-value < 0.05) between the predictor and parents' willingness to vaccinate their children/total number of studies examined the predictor. [b] Number of studies with a negative significant association (p-value < 0.05) between the predictor and parents' willingness to vaccinate their children/total number of studies examined the predictor.

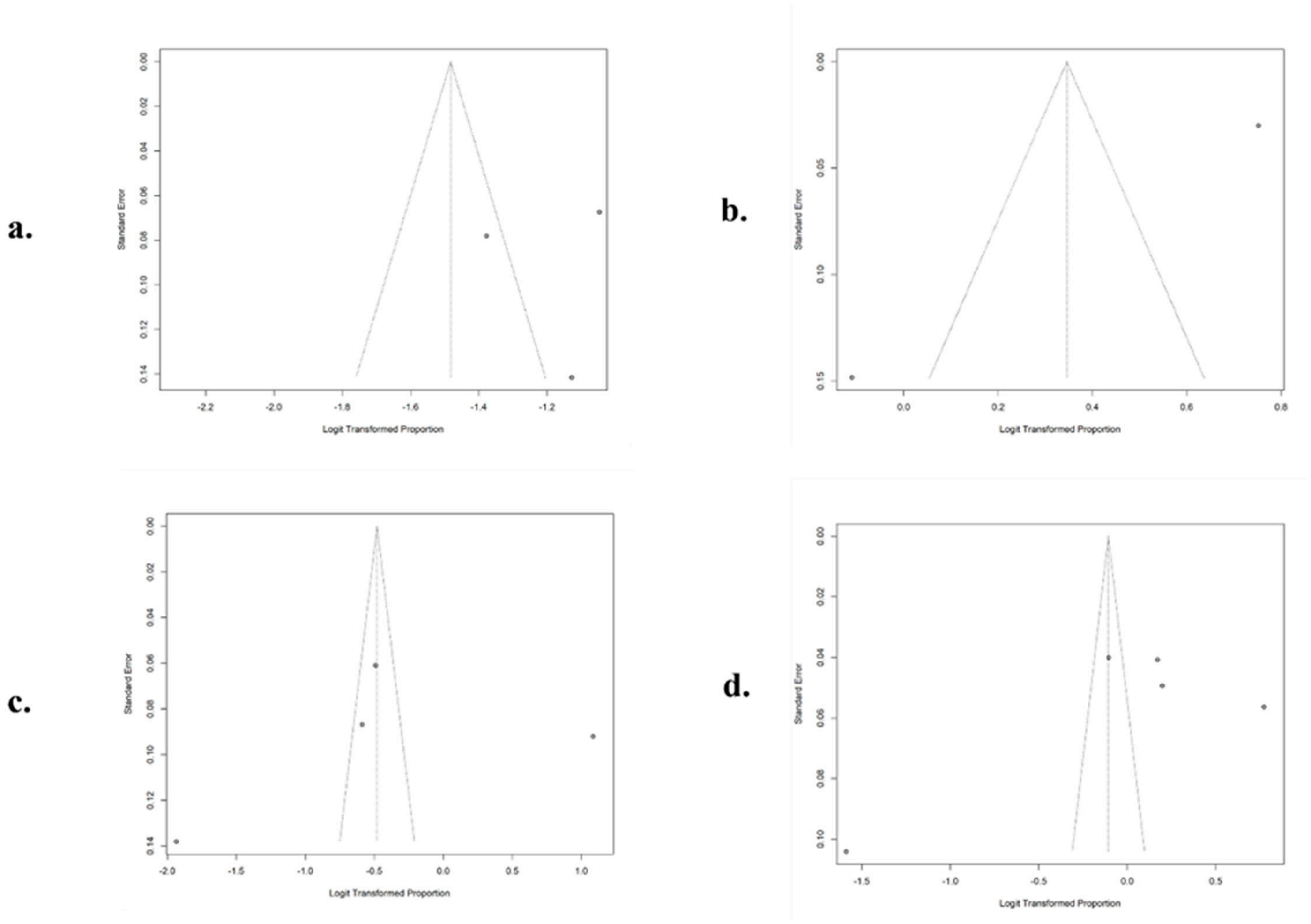

Figure S1. Funnel plots for assessing the publication bias of reported the prevalence of willingness and hesitancy towards vaccination among the included studies. (a. prevalence of hesitancy towards childhood/routine vaccination; b. prevalence of willingness towards childhood/routine vaccination; c. prevalence of childhood/routine delayed vaccination; d. prevalence of willingness towards seasonal influenza vaccination).

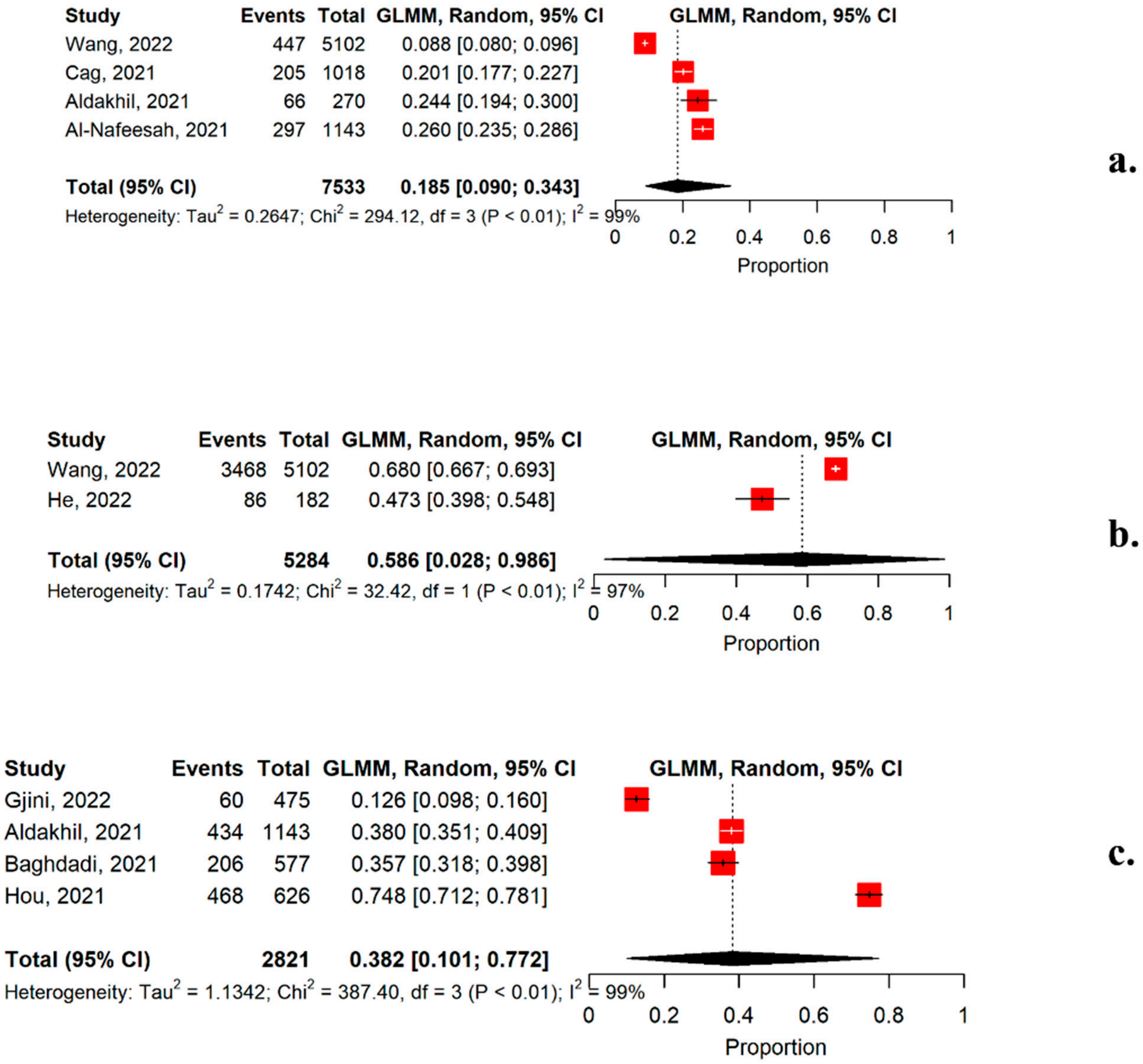

Figure S2. Forest Plot. Pooled prevalence towards childhood/routine vaccination. (a. prevalence of hesitancy towards childhood/routine vaccination; b. prevalence of willingness towards childhood/routine vaccination; c. prevalence of childhood/routine delayed vaccination).

Study	Events	Total	GLMM, Random, 95% CI
Zhou, 2021	996	1456	0.684 [0.659; 0.708]
Seiler, 2021	111	654	0.170 [0.142; 0.201]
Salawati, 2021	1185	2501	0.474 [0.454; 0.494]
Goldman, 2021	1314	2422	0.543 [0.522; 0.563]
Hou, 2021	909	1655	0.549 [0.525; 0.573]
**Total (95% CI)**		**8688**	**0.473 [0.253; 0.705]**

Heterogeneity: $Tau^2 = 0.6165$; $Chi^2 = 437.79$, df = 4 ($P < 0.01$); $I^2 = 99\%$

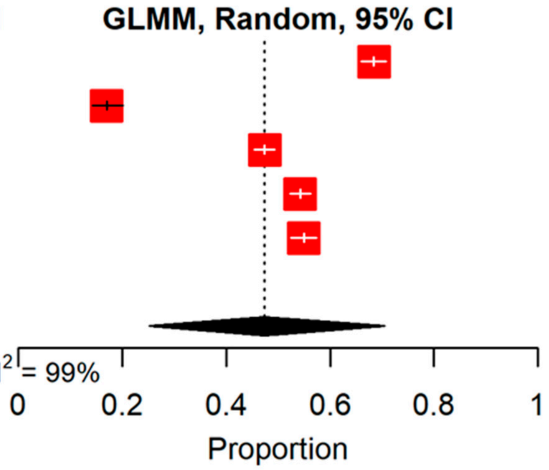

Figure S3. Forest Plot. Pooled prevalence of willingness towards seasonal influenza vaccination.
